# High mobility group A1 protein expression reduces the sensitivity of colon and thyroid cancer cells to antineoplastic drugs

**DOI:** 10.1186/1471-2407-14-851

**Published:** 2014-11-20

**Authors:** Daniela D’Angelo, Paula Mussnich, Roberta Rosa, Roberto Bianco, Giampaolo Tortora, Alfredo Fusco

**Affiliations:** Dipartimento di Medicina Molecolare e Biotecnologie Mediche, Istituto di Endocrinologia ed Oncologia Sperimentale del CNR c/o, Università di Napoli Federico II, Via Pansini 5, 80131 Naples, Italy; Dipartimento di Medicina Clinica e Chirurgia, Università di Napoli Federico II, Naples, Italy; Medical Oncology, ‘G.B. Rossi’ Academic Hospital, University of Verona, Verona, Italy; Instituto Nacional de Câncer - INCA, Rio de Janeiro, RJ Brasil

**Keywords:** HMGA1, Chemoresistance, Colon cancer, Thyroid cancer

## Abstract

**Background:**

Development of resistance to conventional drugs and novel biological agents often impair long-term chemotherapy. HMGA gene overexpression is often associated with antineoplastic drug resistance and reduced survival. Inhibition of HMGA expression in thyroid cancer cells reduces levels of ATM protein, the main cellular sensor of DNA damage, and enhances cellular sensitivity to DNA-damaging agents. HMGA1 overexpression promotes chemoresistance to gemcitabine in pancreatic adenocarcinoma cells through an Akt-dependent mechanism.

**Methods:**

To elucidate the role of HMGA1 proteins in chemoresistance we analyzed resistance to conventional drugs and targeted therapies of human colon carcinoma cells (GEO) that are sensitive to the epidermal growth factor receptor inhibitor cetuximab, and express minimal levels of HMGA1 and cetuximab-resistant (GEO CR) cells expressing high HMGA1 protein levels.

**Results:**

GEO CR cells were less sensitive than GEO cells to cetuximab and 5-fluorouracil. GEO CR cells silenced for HMGA1 expression were more susceptible than empty vector-transfected cells to the drugs’ cytotoxicity. Similar results were obtained with anaplastic thyroid carcinoma cells expressing or not HMGA1 proteins, treated with doxorubicin or the HDAC inhibitor LBH589. Finally, HMGA1 overexpression promoted the DNA-damage response and stimulated Akt phosphorylation and prosurvival signaling.

**Conclusions:**

Our findings suggest that the blockage of HMGA1 expression is a promising approach to enhance cancer cell chemosensitivity, since it could increase the sensitivity of cancer cells to antineoplastic drugs by inhibiting the survival signal and DNA damage repair pathways.

**Electronic supplementary material:**

The online version of this article (doi:10.1186/1471-2407-14-851) contains supplementary material, which is available to authorized users.

## Background

Chemotherapy is one of the most effective tools for the treatment of neoplastic diseases, but it has two relevant drawbacks: 1) it can harm normal cells, and 2) relapse often occurs within 5 years, and recurrent disease is frequently much more resistant to chemotherapy. The advent of new drugs that selectively target specific molecular pathways involved in tumorigenesis or tumor progression, known as “targeted therapy”, has improved patient outcome and survival. However, both conventional chemotherapy and targeted therapies can fail because of acquired drug resistance. Several mechanisms alone or in combination can confer resistance to cancer cells, namely amplification of cell survival signal pathways, increased DNA damage repair, and altered cellular drug uptake, efflux or metabolism [1, 2]. However, each mechanism only partially justifies the lack of response observed in cancer patients. Thus, the identification of other mechanisms mediating drug resistance is a challenge of oncological research.

High Mobility Group A (HMGAs) proteins are small non-histone chromatin factors that bind the minor groove of AT-rich DNA sequences through three N-terminal basic domains called “AT-hooks”. The HMGA family consists of four members: HMGA1a, HMGA1b and HMGA1c (which are encoded through alternative splicing by the *HMGA1* gene) and HMGA2 (encoded by the *HMGA2* gene) [3, 4]. HMGAs are highly expressed during embryogenesis, and low or absent in normal adult tissues. They are overexpressed in almost all human malignant neoplasias, often associated with metastases and a poor prognosis [3]. HMGA proteins play a key role in chemoresistance. Indeed, HMGA2 exhibits dRP/AP site cleavage activity and protects cancer cells from DNA-damage-induced cytotoxicity during chemotherapy [5]. HMGA1 overexpression promotes chemoresistance to gemcitabine in pancreatic adenocarcinoma cells *in vitro* through an Akt-dependent mechanism. Moreover, HMGA1-silencing promotes gemcitabine-induced cytoxicity and reduces tumor growth *in vivo* in a nude mouse xenograft model of pancreatic cancer [6].

Our group also demonstrated the involvement of HMGAs in the pathway of Ataxia-Teleangiectasia-Mutated (ATM) protein, the main cellular sensor of DNA damage. We demonstrated that HMGA proteins positively regulate ATM expression and the inhibition of HMGA1 expression through an antisense approach drastically decreases cellular levels of ATM in anaplastic thyroid cancer (ATC) cells, resulting in increased sensitivity to genotoxic agents [7].

To determine the role of HMGA1 proteins in chemoresistance we have analyzed the resistance to antineoplastic drugs of (i) the human colon carcinoma cells (GEO) that are sensitive to the epidermal growth factor receptor (EGFR) inhibitors cetuximab (CTX) and gefitinib, and that express barely detectable levels of HMGA1, and (ii) CTX-resistant GEO (GEO CR) cells that express high HMGA1 protein levels and are generated through *in vivo* continuous treatment with the drug followed by tumor explant and *in vitro* stabilization of the deriving resistant cancer cell lines [8].

## Methods

### Drugs and treatment

Cetuximab was purchased from ImClone Systems.

Doxorubicin and 5-Fluorouracil were purchased from Sigma (Sigma Aldrich, St Louis, MO, USA).

LBH589 was kindly provided by Dr. Caraglia. For ATM inhibition experiments, cells were treated with KU-55933 (Calbiochem) (10 μM) for 1 h before the induction of ATM kinase activity.

### Cell lines, expression vector and transfection

Human GEO and SW48 colon cancer cells and FRO thyroid anaplastic carcinoma cells were from the American Type Culture Collection (Manassas, VA, USA). GEO CR (CTX resistant) cells were established as described previously [9]. Hairpin RNA interference plasmids were from The RNAi Consortium (Sigma Aldrich). The control PLKO.1 plasmid, which has a scrambled non-targeting short-hairpin (sh) RNA sequence, was from SIGMA. FRO shHMGA1, GEO CR shHMGA1 and respective sh NoTargeting control stable clones were generated by transfection of the above indicated plasmids using the Neon™ Transfection System (Life Technologies, Carlsbad, California). The pCEFLHA and the pCEFLHA-HMGA1, vectors are described elsewhere [10]. GEO pCEFL-HA, GEO-HMGA1, SW48 pCEFL-HA and SW48-HMGA1 cells were generated by transfection of the above-indicated plasmids using the Neon™ Transfection System.

Cells were transfected using Neon™ Transfection System (Life Technologies, Carlsbad, California) under the following conditions:

FRO: Pulse voltage (v): 1450, Pulse Width (ms): 10, Pulse number: 3;

GEO and SW48: Pulse voltage (v): 1300, Pulse Width (ms): 30, Pulse number: 1.

After transfection, stable clones were selected by exposure to 1 μg/ml of puromycin (GEO CR and FRO) or 800 ng/ml of neomycin (GEO and SW48) in complete medium.

### Protein extraction, western blotting and antibodies

Cells were lysed in lysis buffer containing 1% NP40, 1mM EDTA, 50mM Tris–HCl (pH 7.5) and 150mM NaCl, supplemented with complete protease inhibitors mixture (Roche, Branford, CT, USA). Total proteins were separated by SDS–polyacrylamide gel electrophoresis and transferred to nitrocellulose membranes (Amersham, Piscataway, NJ, USA). Membranes were blocked with 5% non-fat dry milk and incubated with the following antibodies: anti-HMGA1 polyclonal antibody, as previously described [10], anti-ATM S1981p (Rockland, Philadelphia, PA, USA), anti-ATM (Ab91) (Abcam Cambridge, MA) β-actin (Santa Cruz Biotechnology, Santa Cruz, CA, USA), (SC-1615; Santa Cruz), anti-Akt, anti Akt S473p and Phospho-p70 S6 Kinase (Thr389) (Cell Signaling, Beverly, MA, USA), anti Caspase-3 (Santa Cruz), anti-phospho-H2AX (ser 139) (Upstate Biotechnology, Lake Placid, NY, USA).

### RNA extraction and quantitative-RT-PCR

Total RNA was isolated using TRI-reagent solution (Sigma) and reverse transcription was performed according to standard procedures (Qiagen, Valencia, CA, USA). qRT-PCR analysis was performed using the following primers:

HMGA1 Fw: 5′-CAACTCCAGGAAGGAAACCA-3′;

HMGA1 Rv: 5′-AGGACTCCTGCGAGATGC-3;

β-actin Fw: 5′- CCAACCGCGAGAAGATGA-3;

β-actin Rv: 5′-CCAGAGGCGTACAGGGATAG -3.

Primers for β-actin were used to normalize qRT-PCR data. To calculate the relative expression levels we used the 2^-ΔΔCT^ method [11].

### Cell viability assay

Drug-induced cytotoxicity was quantified by MTS (3-(4,5 dimethylthiazol-2-yl)-5-(3-carboxymethoxyphenyl-2-(4-sulfophenyl)-2H-tetrazolium) assay (Promega’s CellTiter® 96 AQueous One Solution, Promega Fitchburg, WI, USA). Cells were seeded in 96-well plates at 5×10^3^ cells per well, then exposed to serial dilutions of the drugs. After 72h absorbance was measured at 490 nm.

### Apoptosis assays

Cells were treated with 5FU or doxorubicin and apoptosis was quantified by measuring Caspase 3/7 activation using the Caspase-Glo 3/7 assay (Promega).

### Comet assay

Cells were treated with CTX and allowed to repair the DNA for 0, 18 and 24 hours and then processed for the COMET assay (Trevigen, Helgerman, CT, USA) following manufacturer’s instructions. Cell images were analyzed using COMET Score (TriTek, Annandale, VA, USA). Comet tail moment was used as the measure of DNA damage. In each experiment, 50 comets were measured *per* experimental point and the mean ± S.D. was reported.

### Statistical analysis

We used two-way analysis of variance to compare inter-group differences. The significance of differences was determined by analysis of variances followed by Dunnett’s test as post hoc test using Graph Pad Prism 5.0. Data are reported as mean ± SD and p <0.05 was accepted as statistically significant.

## Results

### HMGA1 overexpression correlates with increased resistance to drug-induced cytotoxicity

**Figure 1:**
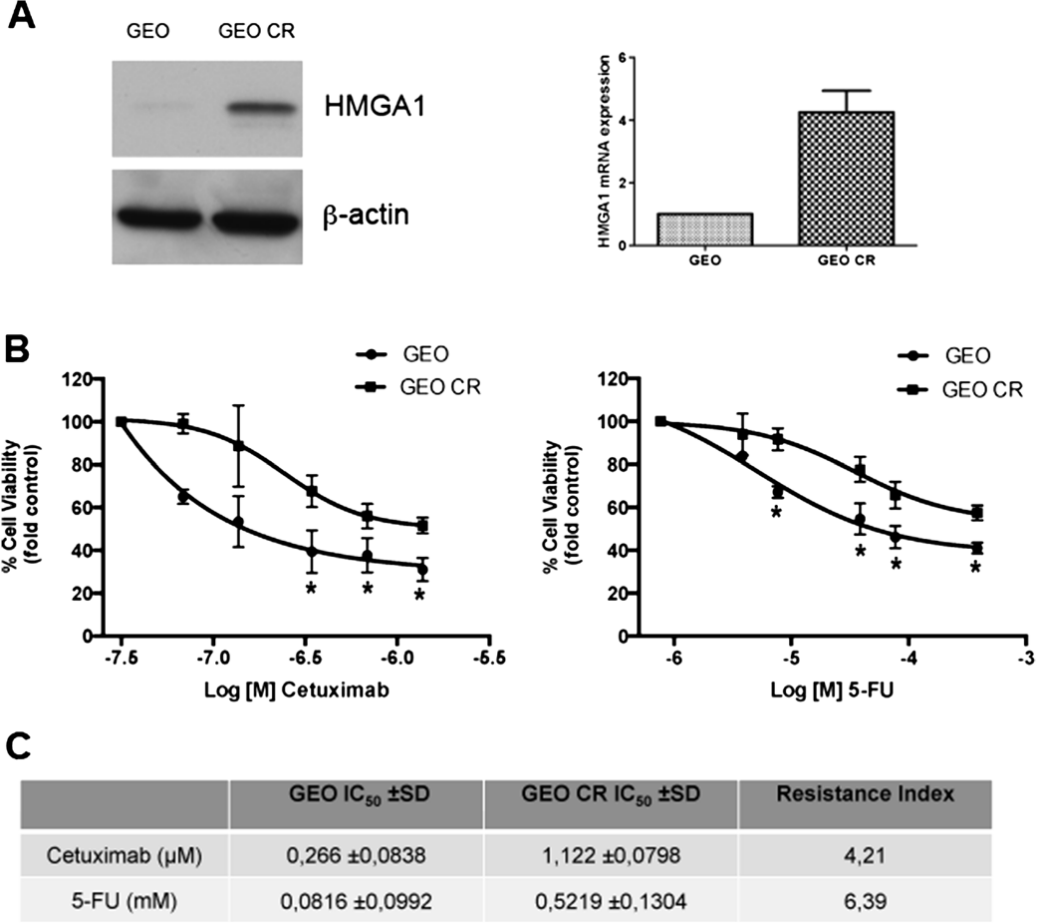
**HMGA1 overexpression is related to resistance to Cetuximab and 5-Fluorouracil in GEO colon carcinoma cells. (A)** Western blot analysis of protein extracts from GEO and GEO CR (Cetuximab Resistant) cells, using HMGA1 and β-actin antibodies. β-Actin was used as loading control (Left Panel). qRT-PCR analysis of HMGA1 mRNA in GEO and GEO CR cells. Relative expression values indicate the relative change in HMGA1 mRNA expression levels between GEO and GEO CR, normalized with β-actin. The error bars represent the mean ± S.D. of three independent experiments performed in triplicate (Right Panel). **(B)** Dose response curve for GEO and GEO CR cells treated with cetuximab (Left Panel) or 5-fluorouracil (Right Panel). Cells were exposed to the indicated doses of drugs and, after 72 h, absorbance at 490 nm was measured using a microplate reader to evaluate cell viability. The results are expressed as percent relative to the control. Values are the mean ± S.D. of three experiments performed in triplicate. Dose response curves were obtained by non-linear curve fitting using GraphPad Prism 5.0. program. Note that the concentration is shown as logarithmic function. *p < 0.05 significance level compared with GEO cells. **(C)** IC50, inhibitory concentration that kills 50% of cell population and Resistance index calculated as IC50 GEO CR/IC50 GEO cell line.

**Figure 2:**
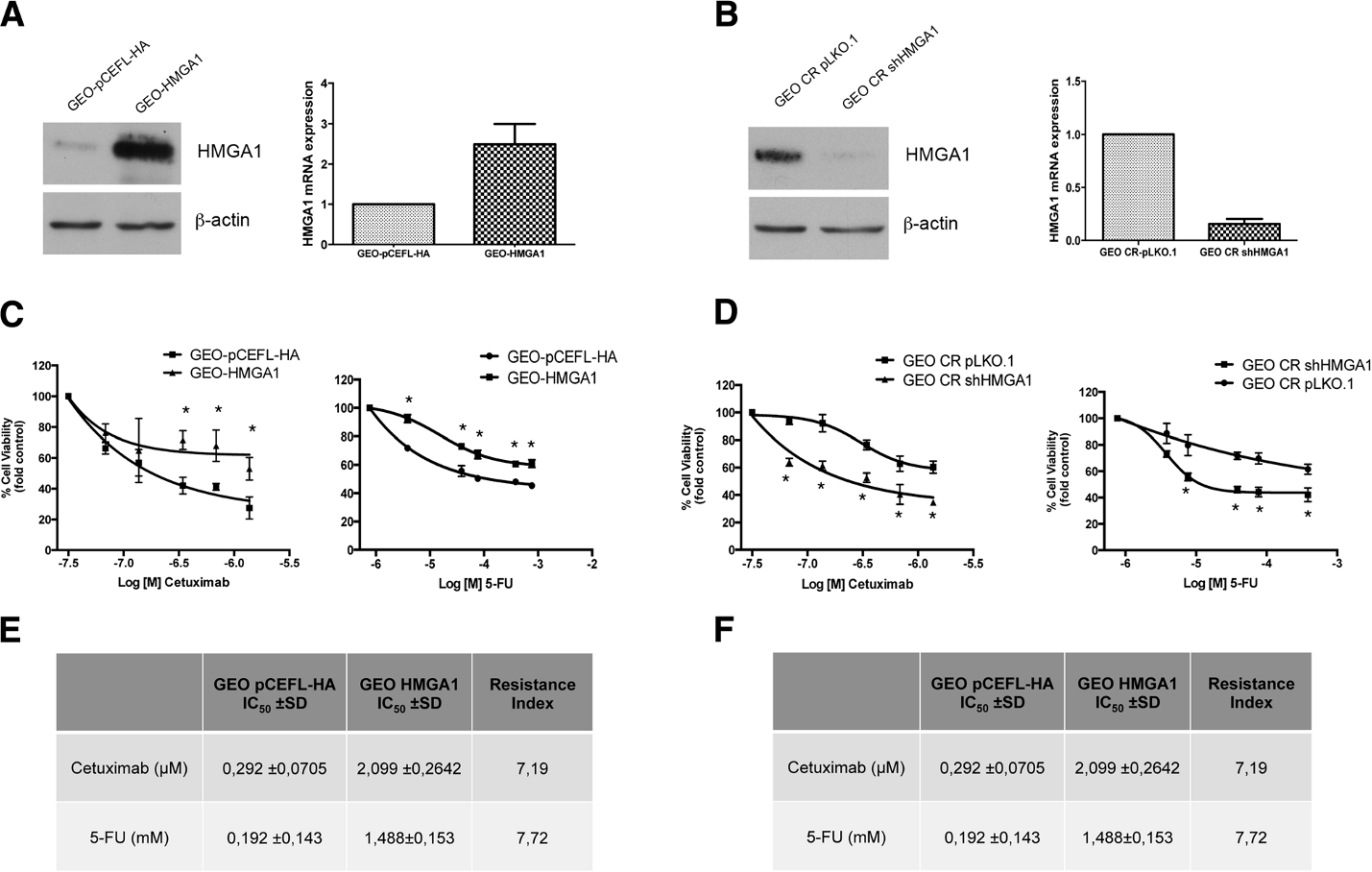
**HMGA1 overexpression or silencing modulate the sensitivity to cetuximab and 5-fluorouracil. (A)** GEO cells stably transfected with a construct expressing the HMGA1 gene (GEO-HMGA1) or with empty vector (GEO pCEFL-HA) and analyzed by western blot using HMGA1 and β-actin antibodies. β-Actin was used as loading control (Left Panel). qRT-PCR analysis of HMGA1 mRNA in GEO pCEFL-HA and GEO-HMGA1 cells. Relative expression values indicate the relative change in HMGA1 mRNA expression levels between GEO pCEFL-HA and GEO-HMGA1 and normalized with β-actin (Right Panel). The error bars represent the mean ± S.D. of three independent experiments performed in triplicate. **(B)** GEO CR stably transfected with a short hairpin RNA targeting the HMGA1 gene (GEO CR shHMGA1) or the empty vector (GEO CR pLKO.1) and analyzed by western blot using HMGA1 and β-actin antibodies. β-Actin was used as loading control (Left Panel). qRT-PCR analysis of HMGA1 mRNA in GEO CR pLKO.1 and GEO CR shHMGA1. Relative expression values indicate the relative change in HMGA1 mRNA expression levels between GEO CR pLKO.1 and GEO CR shHMGA1, normalized with β-actin, assuming that the mean value of GEO CR pLKO.1 was equal to 1 (Right Panel). The error bars represent the mean ± S.D. of three independent experiments performed in triplicate. **(C-D)** Cell viability assay of GEO pCEFL-HA and GEO-HMGA1 **(C)** and GEO CR pLKO.1 and GEO CR shHMGA1 **(D)** cells treated with increasing doses of cetuximab or 5-fluorouracil. Values are the mean ± S.D. of three experiments performed in triplicate.The curves were fitted using nonlinear regression (Graph Pad Prism 5.0. program). The concentration is shown as logarithmic function. *p < 0.05 versus empty vector-transfected cells (GEO pCEFL-HA or GEO CR pLKO.1). **(E-F)** IC50, inhibitory concentration that kills 50% of cell population and Resistance index calculated as IC50 GEO-HMGA1 /IC50 GEO pCEFL-HA cell line **(E)** or IC50 GEO CR pLKO.1/ IC50 GEO CR shHMGA1.

To define the role of HMGA1 in intrinsic and acquired resistance to conventional and biological agents used in cancer therapy, we first analyzed the sensitivity of GEO colon carcinoma cells, showing a very low HMGA1 expression, and of GEO CR cells, expressing abundant HMGA1 levels (Figure 1A), to the antineoplastic drugs CTX and 5-fluorouracil (5FU), which are used to treat colon cancer [8]. As shown in Figure 1C the GEO CR cells were significantly less sensitive than GEO cells to the cytotoxic effect exerted by CTX and 5FU. To determine whether this difference was due to differences in the expression of HMGA1, we stably overexpressed HMGA1 in GEO cells by transfecting them with a construct expressing *HMGA1b* isoform (GEO-HMGA1 cells) (Figure 2A), and silenced HMGA1 expression in GEO CR cells by transfecting them with a short hairpin RNA (shRNA) targeting the *HMGA1* gene (GEO CR shHMGA1 cells) (Figure 2B). As shown in Figure 2C, GEO-HMGA1 cells were less sensitive to CTX and 5FU than GEO cells transfected with empty vector (GEO pCEFL-HA). Consistently, GEO CR shHMGA1 cells were more susceptible than GEO CR transfected with a scrambled no-targeting shRNA (GEO CR pLKO.1 cells).

Moreover, we examined the sensitivity to these drugs of the colon carcinoma cell line, SW48, which expresses low HMGA1 levels, transfected with HMGA1 (SW48-HMGA1) (Additional file 1: Figure S1A). As shown in Additional file 1: Figure S1B, SW48-HMGA1 cells were more resistant to the effect of CTX and 5FU than the empty-vector-transfected SW48 cells (SW48 pCEFL-HA cells).

**Figure 3:**
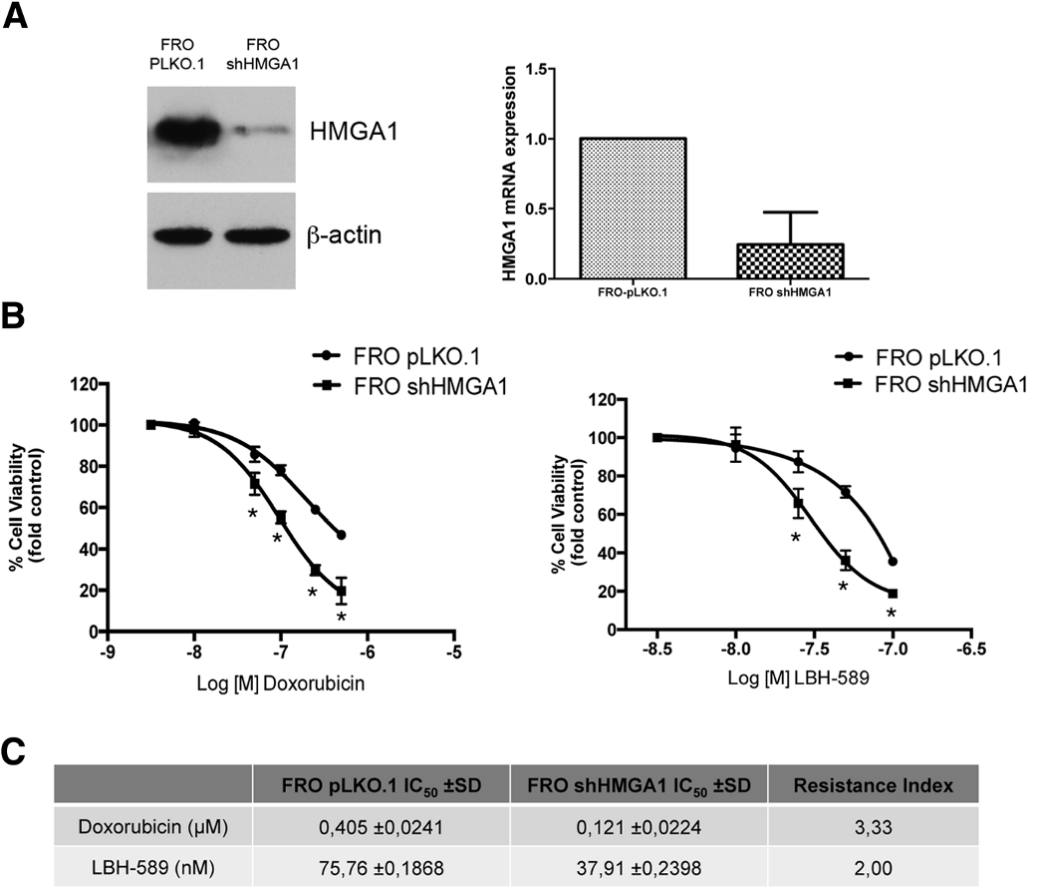
**HMGA1 silencing modulates the sensitivity to doxorubicin and LBH-589. (A)** Western Blot analysis of FRO cells and FRO stably transfected with a short hairpin RNA targeting the HMGA1 gene (FRO shHMGA1) or with empty vector (FRO pLKO.1) using HMGA1 and β-actin antibodies. β-Actin was used as loading control (Left Panel). qRT-PCR analysis of HMGA1 mRNA in FRO pLKO.1 and FRO shHMGA1cells. Relative expression values indicate the relative change in HMGA1 mRNA expression levels between FRO pLKO.1 and FRO shHMGA1cells, normalized with β-actin, assuming that the mean value of FRO pLKO.1 was equal to 1. (Right Panel). The error bars represent the mean ± S.D. of three independent experiments performed in triplicate. **(B)** Cell viability assay of FRO pLKO.1 and FRO shHMGA1 cells treated with doxorubicin (Left Panel) or LBH-589 (Right Panel). Cells were exposed to the indicated doses of drugs and absorbance at 490 nm was measured after 72 hours. Values are the mean ± S.D. of three experiments performed in triplicate. Nonlinear regression lines were calculated using GraphPad Prism5. *p < 0.05 significance level compared with FRO pLKO.1. **(C)** IC50, inhibitory concentration that kills 50% of cell population and Resistance index calculated as IC50 FRO pLKO.1 /IC50 FRO shHMGA1 cell line.

We extended this analysis to FRO cells that derive from a human anaplastic thyroid carcinoma. We transfected these cells with the same construct used to silence HMGA1 in GEO CR cells (FRO shHMGA1) (Figure 3A). We then treated the FRO cells transfected with the empty vector (FRO pKLO.1) and FRO shHMGA1 cells with doxorubicin, that is currently used in the treatment of anaplastic thyroid carcinoma [12] and LBH589 (aka Panobinostat), an inhibitor of HDAC proteins currently undergoing a clinical trial in patients with metastatic medullary thyroid cancer or radioiodine-resistant differentiated thyroid cancer (ClinicalTrials.gov Identifier:NCT01013597). Also in this case, cells overexpressing HMGA1 were more resistant than cells in which the HMGA1 expression was stably silenced (Figure 3B).

**Figure 4:**
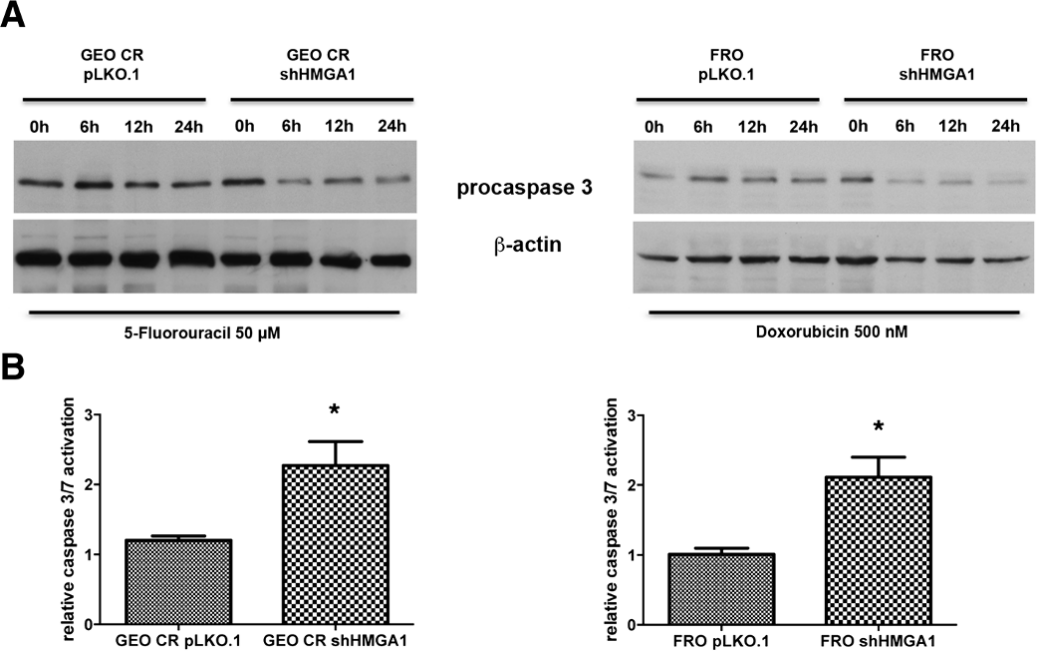
**Silencing of HMGA1 triggers caspase activation in response to antineoplastic drugs. (A)** Cells were treated with 50 μM of 5-fluorouracil (left panel) or with 500 nM of doxorubicin (right panel) and collected at the indicated time points after drug treatment. The uncleaved procaspase-3 was detected by Western blot analysis using a specific antibody. Data were normalized with β-actin expression. **(B)** Colorimetric caspase 3/7 activity assay, performed on GEO CR pLKO.1 and GEO CR shHMGA1 treated with 50 μM of 5-fluorouracil (left panel) and on FRO pLKO.1 and FRO shHMGA1(right panel) treated with 500 nM of doxorubicin and harvested 6 h after the treatment. Values are the mean ± S.D. of three experiments performed in triplicate. *p < 0.05 versus cells transfected with empty vector (GEO CR pLKO.1 or FRO pLKO.1).

We analyzed the apoptotic rate in GEO CR and GEO CR shHMGA1 cells after exposure to 5FU. Drug-induced apoptosis and caspase 3/7 activation were higher in GEO CR shHMGA1 cells than in GEO CR cells (Figure 4A). A similar result was obtained when FRO and FRO shHMGA1 cells were exposed to doxorubicin. Indeed, the apoptotic rate was higher in the FRO shHMGA1 cells in which HMGA1 was silenced than in FRO expressing high HMGA1 levels. Interestingly, procaspase 3 expression decreased earlier in GEO CR shHMGA1 and FRO shHMGA1 cells than in the cells overexpressing HMGA1 after treatment with 5FU and doxorubicin, respectively (Figure 4B).

### Overexpression of HMGA1 correlates with activation of the prosurvival pathway

**Figure 5:**
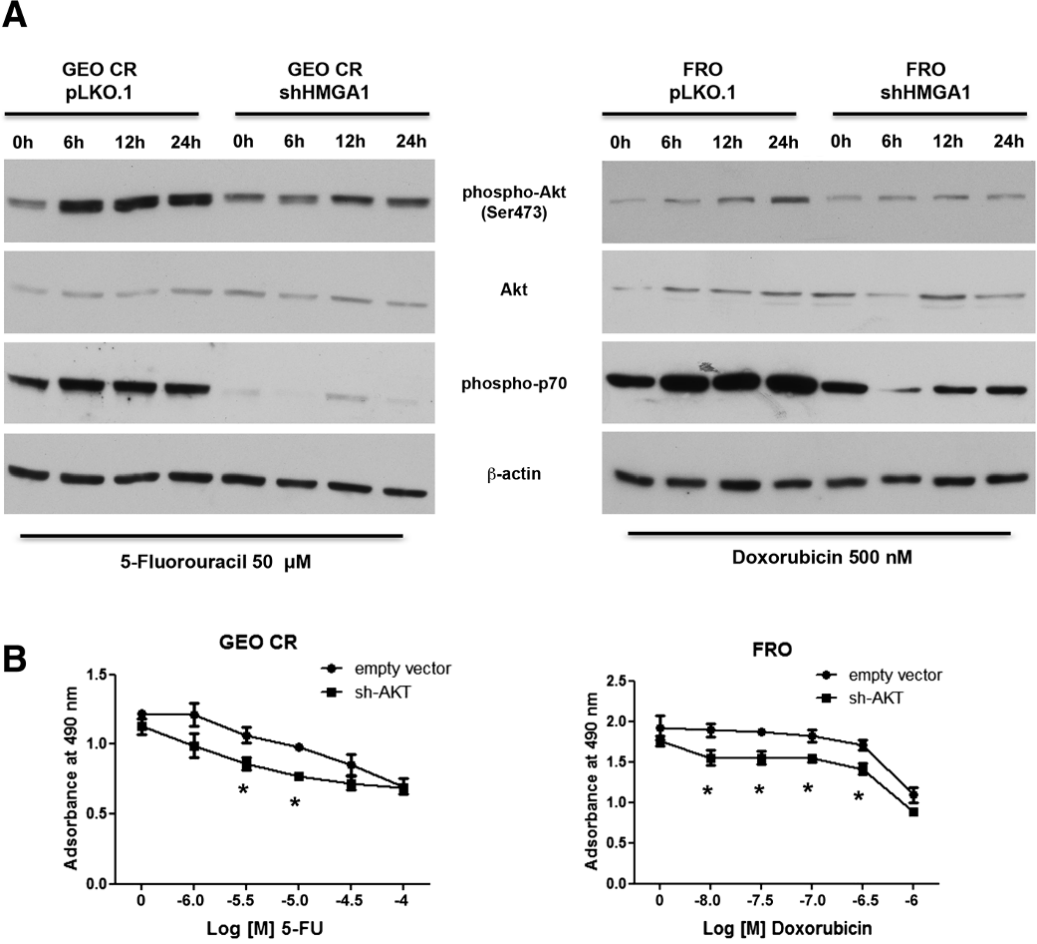
**Overexpression of HMGA1 correlates with the activation of prosurvival pathway. (A)** Cells were treated with 50 μM of 5-fluorouracil (left panel) or with 500 nM of doxorubicin (right panel) and collected at the indicated time points after drug treatment. Protein extracts were analyzed western blot to evaluate Akt and p70 phosphorylation. Total Akt levels are also reported. β-Actin was reported as western blot loading control. **(B)** Cell viability curve of GEO CR and FRO transfected with a short hairpin RNA targeting the Akt gene or with a control short hairpin RNA and, after 24 hours of transfection, treated with the indicated doses of 5FU or doxorubicin. Values are the mean ± S.D. of three experiments performed in triplicate. *p < 0.05 versus cells transfected with empty vector (GEO CR pLKO.1 or FRO pLKO.1).

Resistance to gemcitabine in pancreatic adenocarcinoma cells is mediated by overexpression of HMGA1 through an Akt-dependent mechanism. Indeed, the expression of a dominant Akt construct abrogated the HMGA1-induced increase in chemoresistance to gemcitabine [6]. Therefore, we analyzed the effect of HMGA1-silencing on the Akt pathway in GEO CR cells treated with 5FU. As shown in Figure 5, levels of phospho-Akt and of its downstream effector phospho-p70 were lower in 5FU-treated GEO CR shHMGA1 cells than in GEO CR cells overexpressing HMGA1. Conversely, total Akt protein levels remained unchanged. The same results were obtained in FRO and FRO shHMGA1cells treated with doxorubicin (Figure 5A).

Subsequently, to define the role of the PI3-K/Akt pathway in the antineoplastic drug resistance of cancer cells overexpressing HMGA1, we silenced Akt with a shRNA in GEO CR and FRO cells overexpressing HMGA1. We then treated these cells with increasing doses of 5FU or doxorubicin, and assessed their viability 72 hours later. As shown in Figure 5B, inhibition of Akt protein synthesis reversed the chemoresistance induced by HMGA1 overexpression. Thus, HMGA1-induced resistance depends on Akt signaling.

### Overexpression of HMGA1 correlates with activation of the DNA-damage response

**Figure 6:**
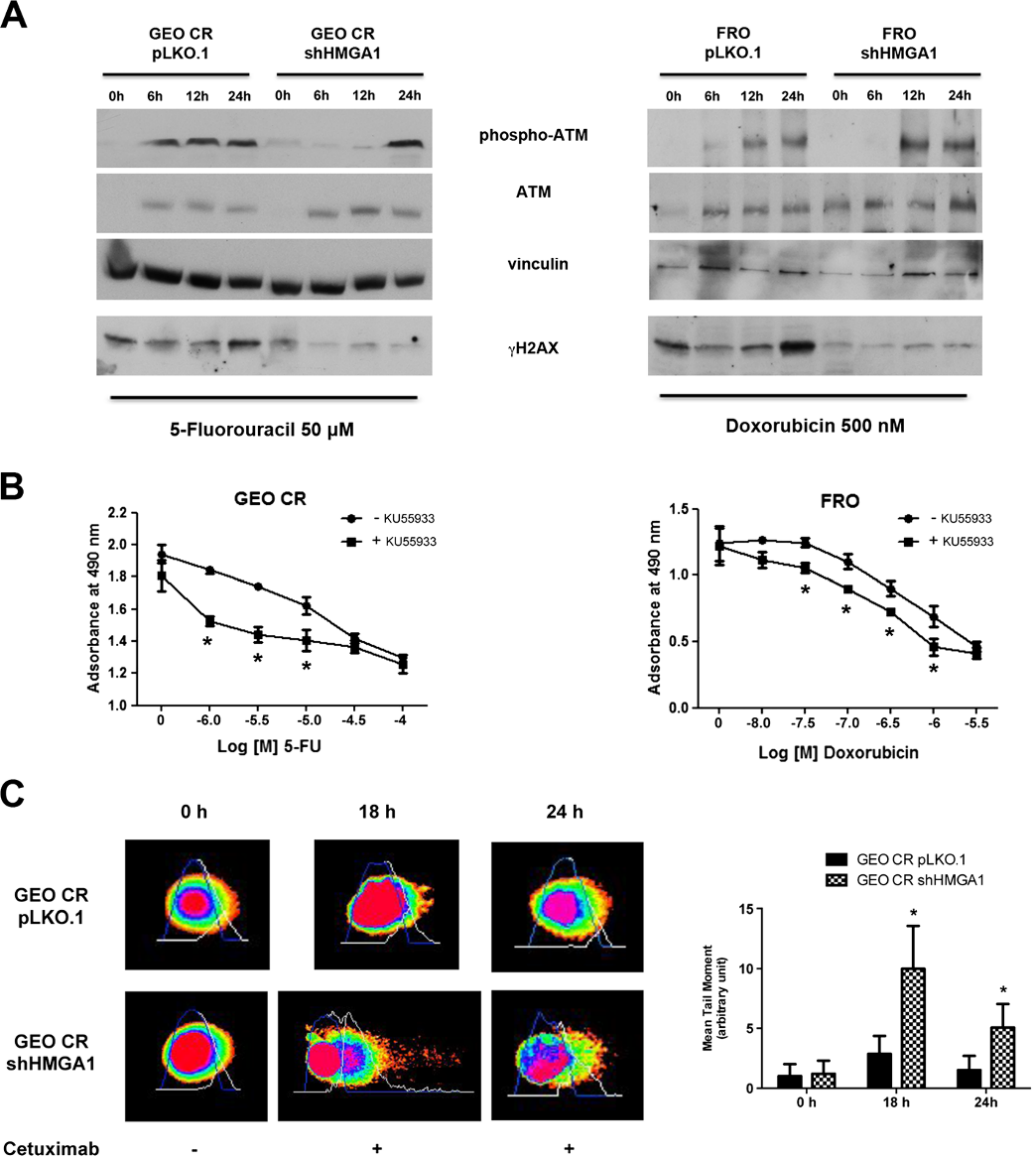
**Overexpression of HMGA1 correlates with the activation of DNA-damage response. (A)** Cells were treated with 50 μM of 5-fluorouracil (left panel) or with 500 nM of doxorubicin (right panel) and collected at the indicated time points after drug treatment. Protein extracts were analyzed western blot to evaluate ATM and H2AX phosphorylation. Total ATM levels are also reported. For H2AX loading control see the β-actin panels in Figure 4. **(B)** Cell viability curve of GEO CR and FRO cells pretreated or not with 10 μM of KU-55933 for 1 hour, and then with increasing doses of 5FU or doxorubicin. Values are the mean ± S.D. of three experiments performed in triplicate. **(C)** GEO CR pLKO.1 and GEO CR shHMGA1 cells were treated with 1 μM of CTX, collected after 0, 18 and 24 hours of treatment and processed for comet assay. Comets were stained with SYBR Green, visualized by fluorescence microscopy and analyzed by COMET Score software. A representative comet for each experimental point is shown in the left panel. Right panel shows the quantitative analysis using the COMET Score software. *p < 0.05 significance level compared with GEO CR pLKO.1.

We previously demonstrated that HMGA proteins enhance cancer cell resistance to genotoxic agents by promoting ATM expression and the cellular response to DNA damage, thereby shifting ATM signaling from cell death to cell survival [7]. Therefore, we analyzed the phosphorylation status of ATM and its downstream effector γH2AX in GEO CR cells treated with 5FU. As shown in Figure 6A, phospho-ATM and phospho-H2AX levels were lower in the 5FU-HMGA1-silenced cells than in 5FU-treated GEO CR cells. Similarly, phospho-ATM and phospho-H2AX levels were lower in FRO shHMGA1 cells treated with doxorubicin than in FRO cells.

To verify that the absence of HMGA1 expression results in accumulation of DNA damage and enhanced sensitivity to antineoplastic drugs, we pretreated cells overexpressing HMGA1 with KU-55933, a specific inhibitor of ATM, and, 1 hour later, we treated them with increasing doses of 5FU or doxorubicin. The cytotoxic effect of the chemotherapeutic agents was higher in GEO CR and FRO cells treated with KU-55933 than in cells treated with 5FU or doxorubin (Figure 6B).

It is known that EGFR stimulate DSB repair after irradiation or activation by its ligands [13] and that the ability of tumor cells to repair DNA damage is reduced following EGFR blockade with Cetuximab [14]. Then, to test whether HMGA1 was able to affect DSB repair after CTX exposure, GEO CR cells expressing or not HMGA1 were treated with CTX, and a comet assay was performed to evaluate the DNA-repair ability following drug exposure. Cells were collected after 0, 18 and 24 hours of treatment and the amount of damaged DNA in each cell type was analyzed evaluating the comet tail moment as a measure of the DNA damage. Interestingly HMGA-silenced cells displayed higher levels of DNA damage after exposure to CTX for 18 hours, compared with GEO CR empty vector. Moreover, after 24 hours HMGA-overexpressing cells were able to almost completely repair the damage, while HMGA-silenced cells still showed significant levels of damaged DNA (Figure 6C).

These data suggest that DSB repair ability is affected by the presence of HMGA1 proteins also after CTX treatment.

## Discussion

Overexpression of *HMGA1* and *HMGA2* is a general feature of experimental and human malignancies and their overexpression is often correlated with aggressiveness, resistance to conventional anti-cancer therapies and poor prognosis [3].

In this study we have evaluated the role of HMGA1 proteins in resistance to both conventional and biological antineoplastic drugs. We found that enforced overexpression of HMGA1 in human colon carcinoma cells GEO, sensitive to CTX and expressing low levels of HMGA1, promoted resistance to CTX and 5FU. Conversely HMGA1-silencing on GEO CR cells abrogated resistance to the above indicated drugs. Similar findings were obtained with FRO cells expressing high HMGA1 levels treated with doxorubicin and LBH589. Accordingly, the HMGA1-silenced cells displayed a higher apoptotic rate and caspase 3/7 activation after exposure to 5FU or doxorubicin. This result is consistent with our previous data showing that the block of HMGA1 expression obtained by using an adenovirus carrying the HMGA1 cDNA in antisense orientation leads thyroid carcinoma cells to apoptotic death [15].

To determine the mechanisms by which HMGA1 promotes drug resistance, we analyzed the Akt-dependent prosurvival signaling. Indeed, previous findings demonstrate that HMGA1 overexpression mediates gemcitabine resistance in pancreatic adenocarcinoma cells through an Akt-dependent mechanism [6], and over-activation of Akt pathway is a poor prognostic factor in cancer [16]. Here, consistent with previous results, we report that HMGA1 silencing inhibits activation of the Akt pathway after treatment with 5FU or doxorubicin.

We next investigated whether the reduced sensitivity of the HMGA1-overexpressing cells was associated with enhancement of the DNA-damage response, since we previously demonstrated that HMGA1 positively regulates cellular levels of ATM in anaplastic thyroid cancer cells so causing reduced sensitivity to genotoxic agents [7]. Functional interactions have been identified between ATM and growth factor-mediated signaling [17]. In fact, ATM is a nuclear protein kinase that functions as a signal transducer in response to DNA damage, but has also a cytoplasmic localization mediating the activation of Akt through a growth factor-mediated signaling pathway [18]. Therefore, the decreased ATM levels in the presence of HMGA1 overexpression might increase pro-survival Akt signaling after treatment of the cancer cells with the antineoplastic drugs.

It has been reported that stimulation of EGFR after irradiation or activation by its ligands, such as EGF or TGFalpha activate DSB repair [13]. The ability of tumor cells to repair DNA damage is reduced following EGFR blockade with Cetuximab [14], and recently it has been demonstrated that the combination of EGFR inhibition and DNA damage-induced therapy increases *in vitro* and *in vivo* response of human tumor cells [19].

Here, we report that the ability of GEO CR cells, resistant to CTX, to repair DNA damage is reduced in absence of HMGA1 proteins. These data support the idea that HMGA1, inducing an overactivation of DNA damage response, makes less effective the blockade of this pathway by CTX. This mechanism could act in combination with the overactivation of PI3K/AKT prosurvival pathway, widely reported upregulated in CTX-resistant cells [20].

## Conclusions

In conclusion, our findings suggest that the block of HMGA1 proteins could increase the sensitivity of cancer cells to antineoplastic drugs by inhibiting the survival signal and DNA damage repair pathways, the overactivation of which is a hallmark of resistance to anticancer therapies and a poor prognostic factor in cancer progression. Therefore, the targeted suppression or inactivation of HMGA1 could be a potential therapeutic strategy with which to increase chemosensitivity in cancer cells.

## Electronic supplementary material

Additional file 1: **HMGA1 overexpression in SW48.** (TIFF 335 KB)
