# Supplementary figures and images for: High mobility group A1 protein expression reduces the sensitivity of colon and thyroid cancer cells to antineoplastic drugs
Source: BMC Cancer. 2014 Nov 20;14:851. doi: 10.1186/1471-2407-14-851 (PMC4247615; doi:10.1186/1471-2407-14-851)

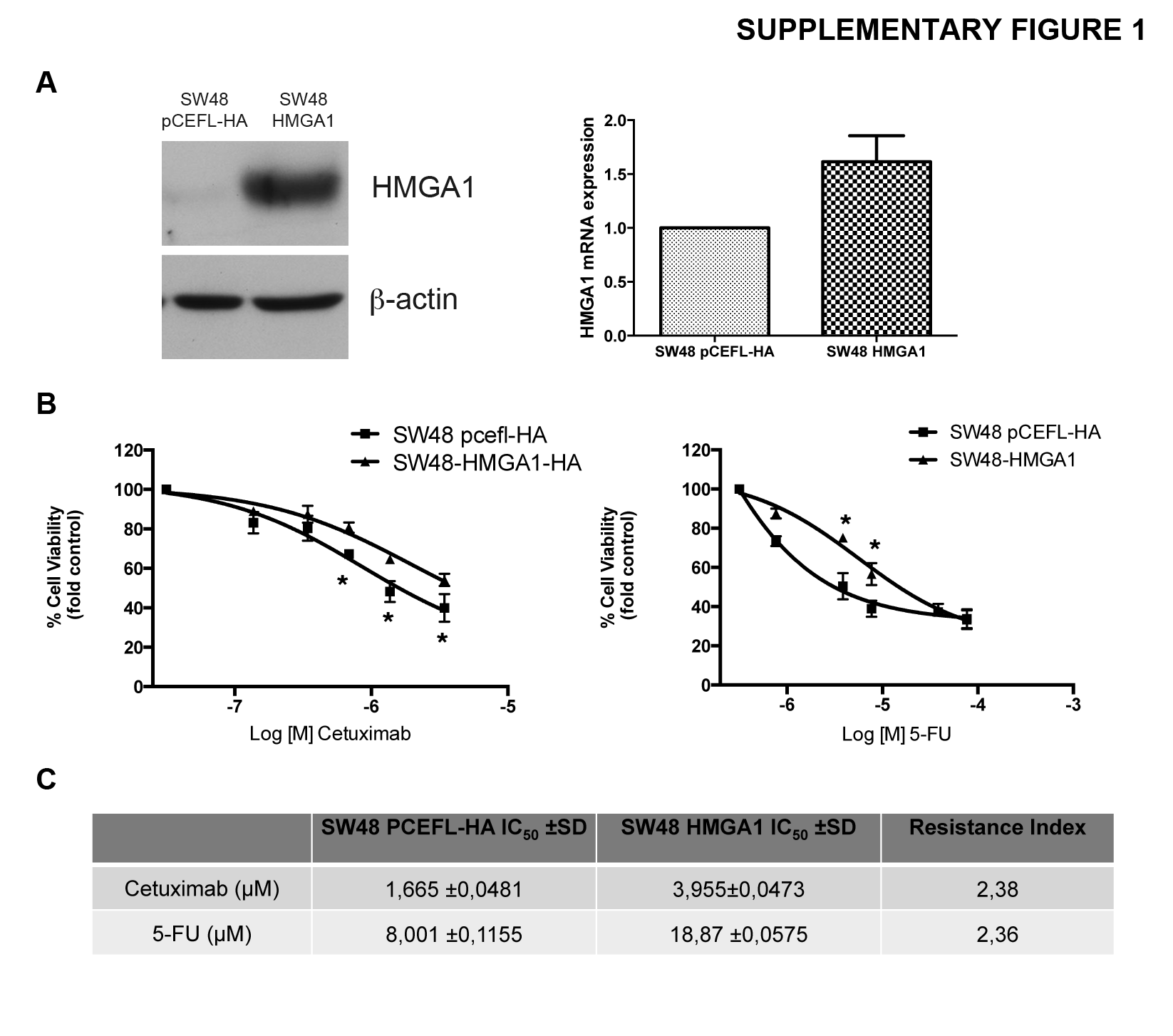

Supplement: Supplementary file 1 — Additional file 1: HMGA1 overexpression in SW48. (TIFF 335 KB) [file 12885_2014_5038_MOESM1_ESM.tiff]
